# Application of Capillary Electrophoresis with Laser-Induced Fluorescence to Immunoassays and Enzyme Assays

**DOI:** 10.3390/molecules24101977

**Published:** 2019-05-22

**Authors:** Binh Thanh Nguyen, Min-Jung Kang

**Affiliations:** 1 Molecular Recognition Research Center, Korea Institute of Science and Technology (KIST), Seoul 02792, Korea; 614007@kist.re.kr; 2 Division of Bio-Medical Science and Technology (Biological Chemistry), Korea University of Science and Technology (UST), Daejeon 34113, Korea

**Keywords:** CE-LIF, immunoassay, enzyme assay, chip-based CE-LIF assay

## Abstract

Capillary electrophoresis using laser-induced fluorescence detection (CE-LIF) is one of the most sensitive separation tools among electrical separation methods. The use of CE-LIF in immunoassays and enzyme assays has gained a reputation in recent years for its high detection sensitivity, short analysis time, and accurate quantification. Immunoassays are bioassay platforms that rely on binding reactions between an antigen (analyte) and a specific antibody. Enzyme assays measure enzymatic activity through quantitative analysis of substrates and products by the reaction of enzymes in purified enzyme or cell systems. These two category analyses play an important role in the context of biopharmaceutical analysis, clinical therapy, drug discovery, and diagnosis analysis. This review discusses the expanding portfolio of immune and enzyme assays using CE-LIF and focuses on the advantages and disadvantages of these methods over the ten years of existing technology since 2008.

## 1. Introduction

Capillary electrophoresis (CE) has become an important tool in the era of separation since its first introduction by Jorgenson and Lukacs in 1981 [1]. The traditional technique, slab gel electrophoresis, initially demonstrated the application of electrophoresis, where charged molecules are separated under an applied electric field over the slab. Despite being straightforward and commonly used in numerous biological laboratories, slab gel electrophoresis is generally time-consuming, and has low efficiency and poor automation. Therefore, electrophoresis carried out in an open tubular glass capillary with an internal diameter of 75 μM was a momentous innovation concerning electrophoretic separation and the development of equipment and instrumentation later on [1,2].

Separation by CE can be conducted by several detectors. Presently, a vast number of detectors fall into one of two categories: bulk property or solute property detectors, where absorption detectors are specific to the latter and attribute to major commercial systems. Using UV or UV-VIS absorbance, the CE analysis deals with a universal range of bioanalytes, since most proteins and macromolecules, such as DNA or RNA, can absorb strongly at radiation in the UV or UV-VIS range [3,4,5,6]. Furthermore, the interface of CE to mass spectrometers or surface enhanced Raman spectroscopy (SERS) is rapidly being promoted to be an online tool to identify sample components [7,8,9,10,11].

Among the detection modes, laser-induced fluorescence (LIF) is one of the most sensitive techniques in terms of the determination and detection of a variety of biomolecules. In recent years, CE has been established as an alternative method of conventional gel electrophoresis or in conjunction with high-performance liquid chromatography.

Bioanalysis based on immunoassays and enzymatic assays has gained a reputation in the field of biological studies and applications in pharmaceutical science, biomarker discovery, and clinical therapeutic and diagnostic targets [12,13,14,15,16,17]. A large number of studies have reported the practice of CE in the research of affinity binding between antibodies and antigens, or kinetic activities of different enzymes to further the understanding of many biological events and developing drug targets in the pharmaceutical industry [18,19,20]. A sophisticated analytical instrument for quantitative purposes such as CE-LIF has become emergent in the moving separation field of biomolecules [21,22,23,24,25,26]. Because CE-LIF performances are generally fast, automated, require a small number of samples, and are highly sensitive, they enable the simultaneous separation of various compounds at different sizes under minute records. Especially, CE-LIF can be merged into miniaturized systems that empowers it to be a high throughput, high-speed tool in the analysis of proteins and peptides [27]. Consequently, the use of CE-LIF in bioanalytical assays has drawn significant attention with the publication of numerous papers dealing with the analysis of biomolecules based on the two significant bio-reaction classes: (1) immune reaction and (2) enzyme reaction. The application of CE based on these two reactions has been extensively reviewed, summarized in [28,29,30,31,32,33,34,35,36].

In this article, we discuss the application of CE-LIF technique in the analysis of proteins and peptides, with an emphasis on immunoassays and enzyme assays in the last decade, from 2008 to early 2019. The details of the instrument conditions, method developments, and advances in the CE-LIF-based assay platforms in the biological studies are also reviewed.

## 2. CE-LIF Instrumentation Labeling Strategies for Peptides and Proteins Analysis

### 2.1. Instrumentation and Laser Sources

As suggested by its name, CE-LIF commonly uses lasers as its excitation source. To accomplish low limit of detection (LODs), it is crucial to maximize the signal and minimize stray light from the optical components and Raman scattering from the solvents. Instrumental LIF designs, such as orthogonal, epi-illumination, or sheath-flow cuvette, have been continually developed in line with CE systems to achieve high sensitivity.

LIF sources for peptide and protein analysis strongly link to the use of fluorescent dyes. These fluorophores absorb light energy in the range of 350–650 nm wavelength. In this range, gas lasers are the most common sources: He–Ne laser (543.5, 593.9, 632.8 nm), Ar laser (454.6, 488, 514.4 nm), Kr laser (416, 530.9, 568.2, 647.1 nm) [37]. Diode lasers provide a stable, thermoelectrically cooled, efficient performance and offer a higher frequency [38,39]. While gas lasers require a kilo-volts power, diode lasers typically run on small voltage supplies. Semiconductor diode lasers emit in the near-infrared (NIR) range, which is an attractive setting which offers a lifetime of over 10,000 h and a volatile range of wavelength [40,41,42]. Another emerging source is light emitting diodes (LEDs), which generate monochromatic light and are a source of great potential for fluoresce measurements in the miniaturized system, and have been used successfully for the detection of various biomolecules [43,44,45,46,47]. LED stands for light emitting diode, and they are a semiconductor device that emit visible light under the application of an electric current. The output from an LED can range from red (~700 nm wavelength) to blue-violet (~400 nm wavelength), or even the infrared (IR) region (~830 nm or longer). Covering a broad spectrum of emitting light, LEDs have become a reliable replacement for LIF, resulting in the formation of LEDIF detection (Light Emitting Diode Induced Fluorescence). CE-LEDIF has been used for the separation of various peptides and proteins [48,49,50]. LEDs are promising as they are less expensive, have lower energy consumption, are more stable, and have a longer lifetime compared to lasers.

LIF detectors can be integrated into an existing commercial CE system, or are available as a standalone, which can be fused with a range of commercial CE or HPLC. The principle of an integrated detector system is using a ball lens to level the laser beam onto the capillary window and an ellipsoid mirror glued on the capillary to collect the emitted fluorescence. The system is secured inside a CE cartridge. A standalone LIF detector typically consists of a laser module, a photomultiplier tube, and the optics with filters, and a dichroic mirror used to reflect the laser beam [51,52]. The flexibility of LIF detection settings allows a dynamic change during operations. For example, Dada et al. reported that LIF detectors, in conjunction with two fiber optic beam splitters and two avalanche photodiodes, gave satisfactory results in the determination of biological analytes in a wide dynamic range [53].

### 2.2. Labeling

CE analysis of proteins and peptides typically utilizes the absorbance detection, which takes advantage of the absorbance of peptide bonds in the ultraviolet (UV) region. For example, spectrophotometry of peptide bonds primarily responds at 200 nm wavelength, and amino acids with aromatic rings are responsible for the absorbance peak at 280 nm wavelength. LIF detection has advanced to be one of the most sensitive CE methods, as it is reported to detect the samples at attomole or zeptomole levels. The first and foremost prerequisite for an analyte to be detected by LIF is the need to be natively fluorescent or chemically derivatized with a designed fluorophore, which is excited at an appropriate wavelength responding to a laser source. Because many peptides and proteins do not exhibit sufficient native fluorescence, due to the dearth of intrinsic fluorescent amino acids, such as tyrosine, tryptophan, and/or phenylalanine, the use of derivatized peptides and proteins has become a common practice of LIF. However, the derivatization procedure contains some drawbacks. For example, at low concentrations of analytes, the reaction yield is low, resulting in poorly labeled analytes and high concentrations of fluorescent backgrounds. In addition, it is challenging to achieve accuracy and reproducibility for the labeling method, thus making the derivatization procedure undesirable for certain types of the analyte. Furthermore, another bottleneck with derivatization of complex analytes is the formation of products attached with multiple fluorophores, which produces a multi-peak chromatography, leading to a perplexing quantification. To circumvent these difficulties, the development of derivatization protocols or studies of new fluorescence dyes has been continuously ongoing. Derivatization of peptides and proteins can be performed pre-, on-, or post-column. Each of these methods has advantages and disadvantages depending on the nature of the fluorophore.

**Figure 1:**
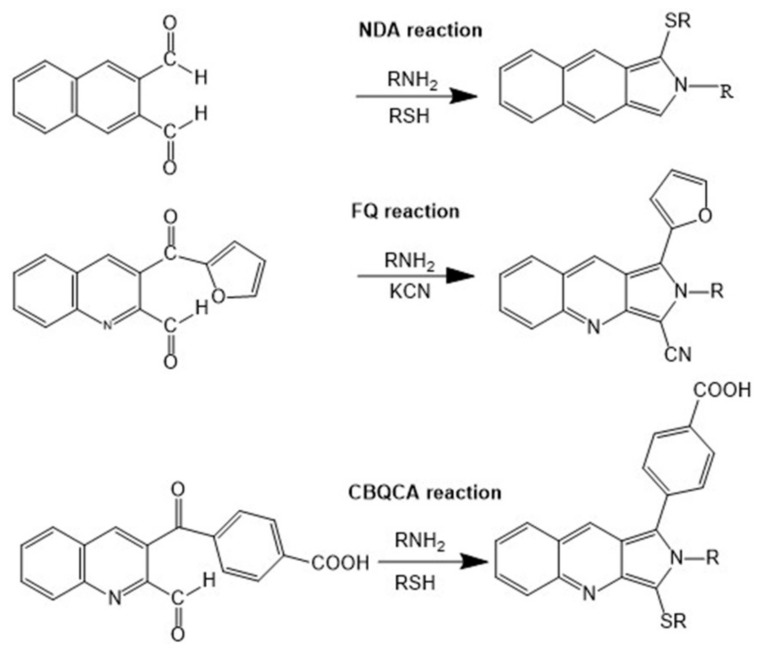
Chemical structures and labeling reactions of naphthalene-2,3-dicarboxylaldehyde (NDA), 5-furoylquinoline-3-carboxyaldehyde (FQ), and (4-carboxylbenzoyl) quinoline-2-carboxaldehyde (CBQA).

Among covalently and fluorescently labeled proteins and peptides, in most case, the dyes can react with primary and secondary amines of amino acids or with thiol groups of cysteine residues. A fluorogenic dye is understood as a precursor of a labeled protein or peptide of interest, but is poorly fluorescent in native form. However, it becomes strongly fluorescent in tagged proteins or peptides after undergoing a chemical or enzymatic reaction. Typical fluorogenic dyes, such as naphthalene-2,3-dicarboxylaldehyde (NDA), 5-furoylquinoline-3-carboxyaldehyde (FQ), and (4-carboxylbenzoyl) quinoline-2-carboxaldehyde (CBQCA), are widely used in LIF detection of the visible region (280–400 nm). LODs obtained are in the range μM–pM [54,55,56,57,58]. The chemical structures and labeling reactions of these dyes are shown in Figure 1. Other covalent labels include 4-Chloro-7-nitro-2,1,3-benzoxadiazole (NBD-Cl), 6-aminoquinolyl-*N*-hydroxysuccinimidyl carbamate (AQC), or near-infrared (NIR) dyes, for example NN382 (LICOR, Inc.).

Besides the utility of fluorogenic dyes, many studies describe fluorescent dyes, such as FITC and rhodamine-based dyes, for LIF detection of longer wavelengths (400–600 nm). Labeling with FITC has become attractive, since it is highly fluorescent [59,60]. However, it is less reactive and efficient at low concentrations of amines with LOD obtained in the range of μM. Nevertheless, rhodamine-based dyes are more reactive compared to FITC, especially if they are activated by the succimidyl ester group, resulting in an efficient reaction with unprotonated amines to form a stable amide bond, thus enabling the LODs in the pM range [61]. For instance, Korchane et al. presented a pre-capillary derivatization strategy of two synthetic transthyretin peptides for the pathology diagnosis, using two fluorogenic dyes (NDA and FQ) and one rhodamine-based tag 5-Carboxytetramethylrhodamine, Succinimidyl Ester (TAMRA-SE). They successfully separated the wild type and mutated type under optimal conditions, with TAMRA-SE labeled derivatives giving the highest resolution, whereas NDA displayed the best detection sensitivity (LOD of 2.5 μM) [62].

Parallel to the use of covalent dyes, noncovalent labels are presented as viable alternatives to reduce the sample handing steps. For instance, idocyanine green greatly fluoresences once it is noncovalently bound to protein, thus allowing the CE-LIF detection at 780 nm [63]. Indigocarmine blue is similar to idocyanine green—it absorbs and emits at 436 nm and 528 nm, respectively. Other noncovalent dyes include Nano Orange, Sypro red, Sypro orange, and Sypro tangerine, which could be detected efficiently under a 488 nm laser. CE-LIF analysis of biopolymers has benefited from using these noncovalent labels, regardless of the slow kinetics of the reactions. While these noncovalent dyes seem to be an interesting approach, the outputs are not conclusive for a broad range of proteins and peptides.

When selecting fluorescent reagents in CE-LIF practice, one needs to consider the following: high quantum yields with low quenching, rapid reaction rates, protein conjugate photostability, and derivatization homogeneity. For example, FQ reacts with ε-amines of lysine to form a stable fluorescent indole derivative, thus generating FQ–protein ligands with high quantum fluorescence efficiencies. Derivatization is accelerated at high temperatures and a moderate pH, and the reaction is simply quenched by dilution with SDS.

Bioassays using native fluorescent proteins and peptides will be excluded from this review, as we focus on the discussion of immunoassays and enzyme assays, as they both rely on the use of fluorescent antigens or antibodies and substrates.

## 3. CE-LIF-Based Immunoassays

Immunoassays using CE techniques—called capillary electrophoretic immunoassays (CEIA) or immunocapillary electrophoresis (ICE)—incorporate an immunological reaction in CE. Unlike other immunoassay methods, CEIA allows the direct visualization of the immunocomplex product formed between an antigen and an appropriate antibody, hence simplifying the interpretation of the results. Compared to traditional methods, such as ELISA, CEIAs are appealing to the study of bioanalytes due to their ease of automation and feasibility of trace amount detection of samples. The concept of performing an immunoreaction on a capillary was first introduced by Nielsen in 1991. Two years later, Schultz and Kennedy demonstrated CE-immunoassays on both competitive and noncompetitive formats to measure insulin using FITC labeled reagents. The combination of the specific immune reactivity with high separation efficiency of CE has rendered the use of this technique to numerous bioanalytes of interest.

Practically, CE immunoassays can be adapted to both homogenous and heterogenous systems. In homogenous assays, analyte, antibodies, and other reacting species are all present in the liquid phase. Two formats that are considered for the combination of CE with immunoreactions in the homogenous system are non-competitive binding and competitive binding, which will be discussed in detail below.

CEIA can be performed on various detection modes, from UV, LIF, and mass spectrometry. CEIA-LIF has become a preferred tool for the analysis of many research groups, due to the high sensitivity and selectivity of the method. The following sections describe the two formats used in CEIA-LIF.

### 3.1. Non-Competitive Binding Format Assay

#### 3.1.1. Principle

Non-competitive CEIA or affinity probe capillary electrophoresis (APCE) involves the quantification of the immunocomplex, which is directly proportional to the number of labeled analytes. Either Ag or Ab needs to be fluorescently labeled. There are two different options for tagged reactants, where Ag* and Ab* indicate labeled reagents:

Ab + Ag* (excess) ↔ Ab-Ag* + Ag* (excess),

Ab* (excess) + Ag ↔ Ab-Ag* + Ab* (excess).

Note that an excess amount of the labeled reagent is added to ensure the complete binding of the analyte present in the reaction mixture. The LIF detector reveals the peak profiles of both the immunocomplex product and excessive labeled Ag or Ab based on their relative differences in size and charges. It is possible to quantify the amount of Ab or Ag in the mixture according to the amount of immunocomplex formed or/and the decreased amount of free labeled agents. Nevertheless, this direct format is limited in use because the binding between small molecules, as with an antigen, and large molecules, as with an antibody, do not significantly vary the electrophoretic mobility of the labeled molecular recognition elements, hence impeding the separation of labeled reagents from the immunocomplex.

#### 3.1.2. Application

The non-competitive CEIA format coupled with LIF detection has been widely used as a part of APCE techniques in the separation of a large variety of biomolecules. The major advantage of non-competitive assays is the commercial availability of labeled antibodies. Liu et al. developed a non-competitive CEIA-LIF to detect alpha-fetoprotein (AFP) in the early diagnosis of primary hepatoma [64], using poly (guanidinium ionic liquid) monolithic material. In this assay, AFP was incubated with an excess amount of fluorescently labeled antibody to form an immunocomplex, and thereafter separated accordingly. Under optimized conditions, their assay performed an LOD of 0.05 μg/L AFP. Compared to other immunoassays to detect AFP, their method exhibited higher sensitivity and larger linear dynamic range, specifically, no purification process, thus shortening the analysis time. By combining a CE-LIF immunoassay with fluorescence polarization, Wang et al. described a method for rapid and sensitive detection of genomic DNA methylation without tedious processes such as the bisulfate conversion, enzyme digestion, or PCR amplification. In this assay, the immunocomplex of methylated DNA was recognized by the fluorescently labeled secondary antibody and separated from unbound antibody by CE-LIF. The analytical performance accomplished an LOD of DNA methylation at 0.3 nM, proving the CE feasibility in the separation of a great diversity of compounds [65]. Besides this, it demonstrated the flexibility of labeling reagents, not only limited to primary antibodies but also applicable to secondary antibodies.

**Figure 2:**
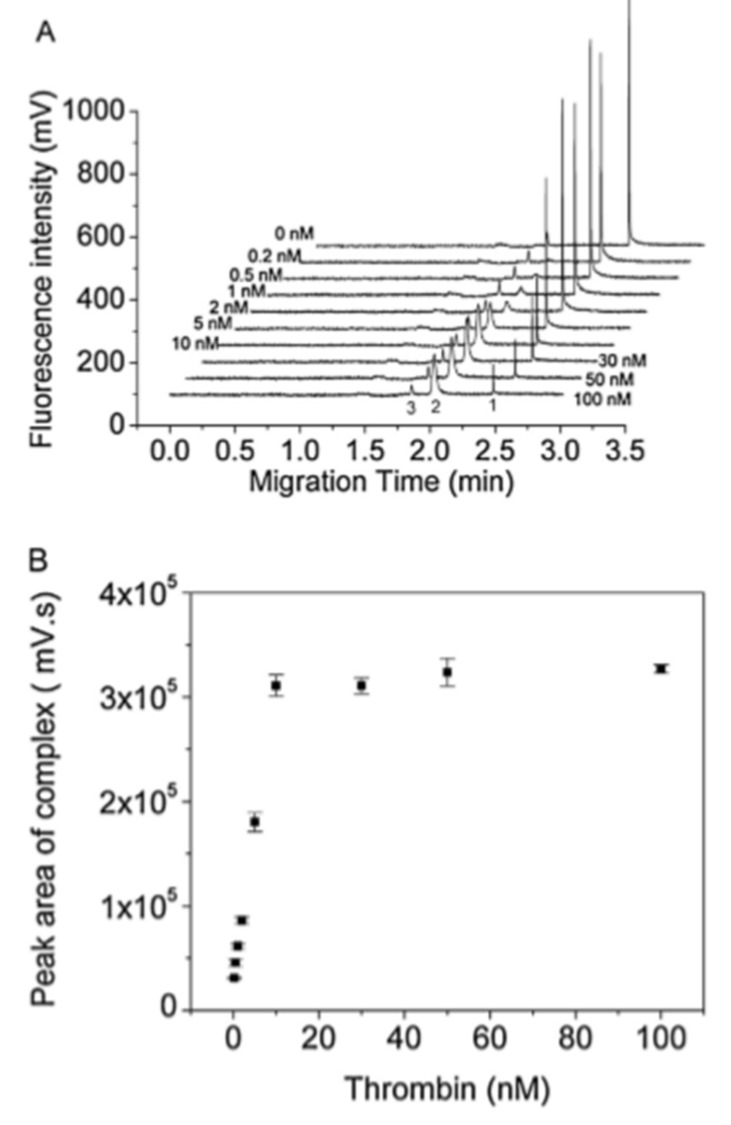
Capillary electrophoresis using laser-induced fluorescence detection (CE-LIF) detection of thrombin using tetramethylrhodamine TMR labeled Toggle-25. (**A**) Electropherograms of Toggle-25-TMR in the presence of varying concentrations of thrombin. (**B**) The relationship between the peak area of complex and the concentrations of thrombin. Reproduced under permission of [66].

As mentioned previously, the direct format is challenging to achieve because of unrecognized electrophoretic mobility change and difficulty in homogenous labeling. As a result, the use of labeled antibodies as detected traces has been restricted. Instead, several research groups are paying attention to the application of aptamers for use as a binding ligand in APCE separation. Aptamers are single-stranded nucleic acids with advantageous features over antibodies, such as high stability, ease of synthesis, and high binding capacity. In CE-LIF, the aptamer is usually labeled with a fluorescent dye. Owing to their smaller molecular weight than proteins (5–15 kDa), the binding of aptamers on larger size proteins can significantly change the ratio of charge to mass of the labeled aptamer. Consequently, the electrophoretic mobility change is significantly improved, enabling the CE separation of immunocomplex and free aptamers much more easily. Hao et al. described a non-competitive CE-LIF-based method to study human thrombin, an essential protein related to the blood coagulation pathway, and successfully detected thrombin in human serum at 0.2 nM using dye-labeled nuclease resistance aptamer Toggle-25 (Figure 2) [66]. Prior to their work, Song et al., reported an affinity probe capillary electrophoresis/laser-induced fluorescence polarization (APCE/LIFP) and achieved the detection of thrombin at low LOD at sub nanomoles/liter [67]. Yi et al. demonstrated a non-competitive CE-LIF assay to detect picomolar concentrations of glucagon and amyline, using labeled mirror-image aptamers called Spiegelmers [68]. The LOD obtained for glucagon was 6 pM and for amylin was 40 pM. These LOD values were lower compared to those obtained from the competitive immunoassay format using rival Ab as affinity ligands in their previous works. The reproducibility of the detection methods for glucagon and amylin (Relative Standard Deviation for peak height, RSD) were <5.8% and <5.3%, respectively.

By using aptamer CE-LIF application, other non-competitive assays have successfully detected several proteins and peptides, including platelet-derived growth factor (PDGF) [69], human immunodeficiency virus reverse transcriptase [69], human immunoglobulin E [69], and recombinant human erythropoietin-α [70]. CE-LIF aptamer separation also enables the simultaneous analysis of multiple proteins in a single run, ranging from various proteins [69] to small analytes [71]. In most aptamer-based affinity CE-LIF assays, DNA aptamers are usually favored, whereas the use of RNA aptamer is still modest. This is due to the poor stability of RNA aptamers compared to DNA aptamers. Especially in the biological matrix, RNA aptamers tend to be quickly degraded. When selecting a non-competitive format immunoassay, one needs to consider several factors to maximize the performance. For instance, only tagged species can produce fluorescence, and these signals should not be interfered with by analytes or sample matrix. The sample matrix or background electrolyte effect should be studied carefully, because they can enhance or reduce the signals which confound the legitimacy of the method. The compilation of the non-competitive format assay is presented in Table 1.

### 3.2. Competitive Binding Format Assay

#### 3.2.1. Principle

In a traditional competitive immunoassay, the amount of one reactant is limited. Similarly, in a CEIA-LIF-based assay, the fluorescent reagent (Ag* or Ab*) competes with non-fluorescent analog (Ag or Ab) to bind with a limited amount of corresponding immunological reactants. The reactions can be formulated as follows:

Ag + Ag* + Ab (limited) ↔ Ab-Ag + Ab-Ag* + Ag + Ag*,

Ab + Ab* + Ag (limited) ↔ Ab-Ag + Ab*-Ag+ Ab* + Ab.

The fluorescently labeled reactant competes with free isotypes in the mixture to form a fluorescent immunocomplex. CE-LIF profiles display two distinct peaks, corresponding to the free labeled reactant and the immunocomplex. The concentration of the antigen (or antibody) is directly proportional to that of the labeled antigen (or antibody), but inversely proportional to that of the immunocomplex.

#### 3.2.2. Application

**Figure 3:**
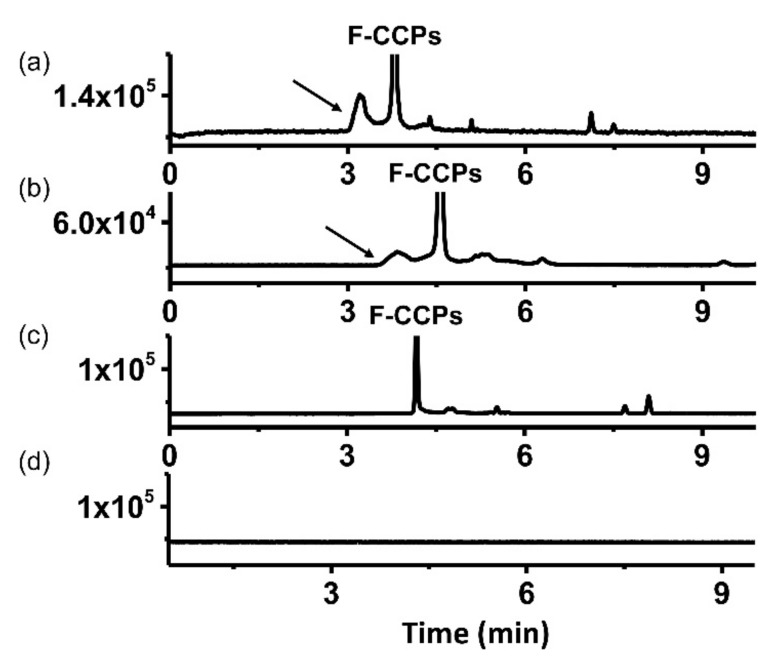
Electropherograms of immunocomplexes of fluorescent cyclic citrullinated peptides (F-CCP) with (**a**) anti-cyclic citrullinated peptides (CCP) antibodies in PBS, (**b**) anti-CCP antibodies in Fetal Bovine Serum, and (**c**) human IgG. (**d**) Electropherogram of patient samples without F-CCP treatment. Arrows indicate the peak from the immunocomplex of F-CCP and anti-CCP antibodies. Reproduced with the permission of [60].

Since being developed, the competitive binding assay has gained a growing reputation and become the most popular format for CE-LIF-based immunoassays. Giovannoli et al. assessed the variables affecting the performances of a competitive CE-LIF assay for the detection of human serum albumin. They showed that the development of the CEIA-based assay was similar to that applied in conventional microplate immunoassays, regarding the interface of peculiarities [72]. The same group also reported another CE-LIF competitive immunoassay to investigate Cry1bAb endotoxin from Bacillus thuringensis with an LOD of 0.5 nM, and allowed the satisfactory recovery (62–98%) of the protein in real samples [80]. In 2018, we successfully developed a quantitative CE-LIF-based assay to detect the antibodies against cyclic citrullinated peptides (CCP) for the diagnosis of rheumatoid arthritis. Our method enabled the quantification of the concentration of anti-CCP antibodies in patient sera, ranging from 0.1–0.4 μg/mL (Figure 3). For the detection of anti-CCP antibodies, our assay achieved a reproducibility within 5% and accuracy ranging between 89% and 103%. Compared to a semi-quantification method, such as ELISA, our CE-LIF-based method outperformed ELISA in terms of specificity and sensitivity [60]. In addition, the analysis was fast, reproducible, and no clean up step was required for intricate sample matrices, making it highly adaptable to other disease diagnoses using biological fluids. In 2015, Liu et al. discussed a simple competitive CE-LIF assay to determine the concentration of norfloxacin in food samples. Norfloxacin is a compound that is widely used in the treatment of gonococcal urethritis, respiratory and skin infections, but is consequently retained in animal-derived foods and leads to public health threats. Compared to the traditional methods such as ELISA and HPLC-MS/MS, their CEIA-LIF displayed high sensitivity, reduced laborious washing steps, and established an efficient quantitative tool for the selective determination of chemical residues in biological matrices. The proposed method obtained an LOD of 0.005 μg/L of norfloxacin, and the RSDs for migration time and peak area of the immunocomplex were 0.17% (intraday) and 3.46% (interday), respectively [73]. Zhang et al. performed a CE-LIF competitive format to effectively detected carbaryl in rice samples, with 14 times greater sensitivity than that of ELISA, which used the same immune-reagents [74]. Several groups also reported the application of CEIA-LIF in the detection of alpha-fetoprotein (AFP) and thyroxine (T4) in human sera, and chloramphenicol in animal-derived foods [75].

Like the non-competitive format, CEIA-LIF competitive assays benefit enormously from the development in the application of various bioanalyses. Zhang et al. introduced a method to determine receptors of PDGF, where the receptor β competed with a fluorescent aptamer in binding to PDGF-BB. The aptamer bound strongly with the β receptor but not the α receptor of PDGF, resulting in a difference in the electrophoretic mobility of PDGF isomers, which allowed the separation of PDGF’s isomers in a single analysis. This is also a significant advantage of CE over MS which cannot distinguish compounds with the same molecular weight. Zhang’s work successfully demonstrated the simultaneous detection of PDGF isomers and their receptors in a single run [76]. Aptamers binding to analytes often leads to signal changes, a mechanism called structure switching. Using this concept, Zhu et al. described a novel APCE strategy dedicated to small molecule detection. They established a pure, high throughput and versatile APCE-LIF in terms of adaptability, generalizability, and capability for multiple detections of small size analytes in a single capillary [81]. Other applications of competitive binding CE-LIF aptamer-based immunoassays can be found in Table 1.

Competitive binding assays have become the most critical platform in CE-LIF-based immunoassays, due to the easier separation of the bound and non-bound labeled reagents. Furthermore, only an analog of the analytes needs to be labeled, which prevents the production of multiple homogeneous preparations of labeled antibodies that are often encountered in the non-competitive format. However, competitive assays tend to have a higher limit of detection and a smaller dynamic range compared to that of non-competitive assays, and more difficulty in distinguishing cross reactivity between species than when a non-competitive format is employed.

### 3.3. Microchip-Based CEIA-LIF

Electrophoresis performed in microchips was first introduced by Manz and Harrison in 1992 [82]. Compared to traditional CE, the integration of microfluidics into the system to manipulate, automate, and analyze the minimum volume of analytes has opened up a new era for high-throughput analysis, a key to industrialized drug discovery. Today, many pharmaceutical companies are screening up to 300,000 or more compounds per screen to produce 100–300 hits. The concept of immobilizing immuno reagents for measuring the bioactivities of drugs adds a dimension to the existing drug discovery paradigm. Numerous reviews have discussed the application of microfluidic devices in the CE system with regards to the time period [83,84,85,86,87]. The previous sections discuss a general homogenous format, where antibodies and antigens are present in the solution phase. The chip-based method represents a heterogeneous format in which either the analyte, antibody, or analog of the binding agents are immobilized onto a solid support [88]. Similar to the homogenous system, immunoassays carried out by CE use both noncompetitive and competitive formats [89,90]. The combination with LIF high selectivity and sensitivity features has drawn attention to microchip-based CE-LIF for use in biological and clinical studies. Phillips and Wellner introduced an immunoaffinity chip-based CE-LIF method to measure the concentration of a brain-derived neurotrophic factor in human skin biopsies [91]. The antibodies were chemically immobilized on a replaceable immunoaffinity disk. Homogenates obtained from micro-dissected human skin samples were subject to the immunoaffinity inserts, in which the analyte of interest was captured, followed by fluorescent labeling with a red-emitting laser dye, before being detected by LIF. Compared to conventional immunoassays, this chip-based CEIA-LIF demonstrated a good correlation. In addition, this system has the potential to be modified into a portable unit for clinical or biomedical screening. The same group later designed another microchip-based CE-LIF device to study chemokines in samples of neuro inflammatory premature infants. Using similar strategies as in the previously described method, the system took only two minutes to successfully isolate six analytes in a single run. The method compared well to a commercial ELISA. In addition, the CE chip was more reliable, and required significantly fewer samples, a crucial criterion when studying newborns [92]. Very recently, this group published a chip-based CE-LIF immunoassay to study inflammatory mediators in newborn dried blood spot samples [93].

The application of chip-based CE immunoassays to multi-analysis was demonstrated by the Shi et al. study on multiple tumor markers [94]. The detection of carcinoma antigen 125 (CA125) and carbohydrate antigen 15-3 (CA15-3) was based on an offline noncompetitive immunoreaction of CA125 and CA15-3 with FITC-labeled monoclonal antibodies. Subsequently, the microfluidic multiplexed channel coupled with the LIF detector allowed the separation of CA125 and CA15-3 contents in the sera of cancer patients, as well as healthy cohorts. In contrast to various traditional immunoassays determining CA125 and CA15-3, such as immunoradiometric assays or immunofluorometric assays, the chip device enabled a rapid, small reagent consumption, and simple operation performance.

Chip-based CEIA-LIF relies on the sources in which the reagents are immobilized. For the methods based on immobilized antibodies, the conditions should be considered cautiously in order to release the captured analytes, which sometimes remain challenging because some antibodies with strong affinity binding are utilized for such work. However, because of its strong binding capacity, this approach is useful to isolate and concentrate trace substances before analysis by CE. It is necessary to ensure there is no irreversible damage to the immobilized antibodies on the chip. For the methods based on immobilized analogs of the analyte, to create the immobilized support, the analyte may need to be derivatized with appropriate functional groups, which help the immobilization within the CE wall. Moreover, this procedure should not interfere with the interaction between antibodies and antigens. For a small-size immobilized tracer, a spacer arm must be included to link the analog and the device, to enable accessibility to antibodies for binding.

## 4. CE-LIF-Based Enzymatic Assays

Since first being introduced by Banke et al. [95] to detect the enzyme activity of alkaline protease, CE coupled with enzymatic reaction has been widely applied to study and characterize enzyme-catalyzed analysis. Enzymes regulate nearly all physiological chemical reactions in living organisms and catalyze all aspects of cell metabolism. The extensive studies of enzymes as current drug targets have encouraged researchers to seek effective methods to characterize enzyme activities and understand their roles in human diseases. Such activities include enzyme kinetics, enzyme substrate identification, enzyme inhibitor screening, and enzyme-mediated metabolic pathways, and these aspects of enzyme-related analysis have been largely studied by CE methods, for example, those summarized in [96,97,98,99]. A typical CE-based enzyme assay starts with small amounts of the substrate, enzyme, and/or inhibitors. When the enzymatic reaction is completed, the CE can directly detect and separate the components of interest without the requirement of additional coupling factors, such as co-enzyme, which reduce the sample complexity and the likelihood of fall positives, especially when it comes to the studies of enzyme inhibition. In general, CE-based enzyme assays can be grouped into two main categories: (1) pre-capillary (offline) assays, in which the reaction is performed outside the capillary before being injected into the system, and (2) in-capillary (online) assays, in which the enzymatic reaction takes place inside the capillary, where injection, mixing, reaction, separation, and detection are integrated into a single column. Details of these two categories are discussed in this section.

### 4.1. Off-Column (Pre-Column) Enzymatic Assays

In off-column assays, or so-called pre-column enzymatic assays, the reaction is carried out in a separate system. Enzymes, substrates, and/or inhibitors are mixed and incubated, and the sample is subjected to CE analysis, where the substrates’ and/or inhibitors’ activities are determined. The capillary only functions as a separating channel. Because this format is easy to monitor—due to the ability of enzymes to catalyze under straightforward conditions—offline methods have been applied to enzyme inhibitor screening and drug metabolism studies for years. However, there are some drawbacks to the offline mode. Firstly, CE consumes only small volumes of the sample. However, since it is performed in a vial, the reaction requires much larger volumes of both enzymes and substrates to undergo the reaction and multiple steps may be needed to operate, such as adding the quenching reagents to terminate the enzymatic reaction, or changing the reaction conditions before the analysis by the CE system. This leads to the waste of reagents, especially for expensive enzymes, and the addition of strong acids during the quenching step may contribute to the peak distortion later. Moreover, interference between the incubation buffer and separation background electrolyte may result in EOF variability, peak broadening, and eventually low sensitivity and reproducibility of the method. Therefore, the LIF mode has offered to enhance the sensitivity for the detection based on pre-capillary methods.

### 4.2. Application

**Figure 4:**
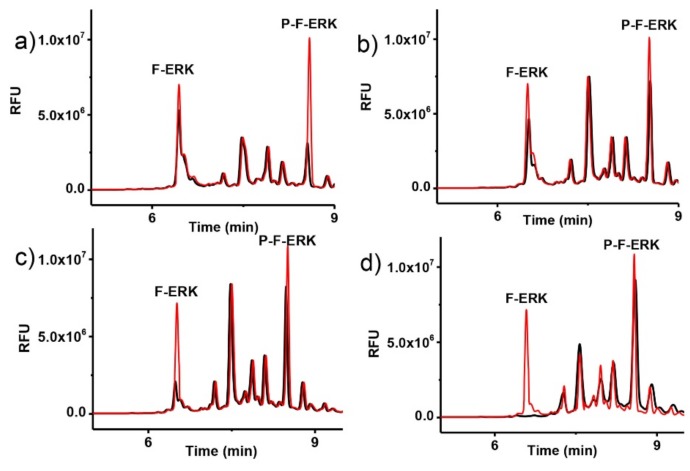
Time course study of enzymatic reaction. Extracted media from gastric cancer MKN-1 cells treated with 2 µg/mL F-ERK, 0.01 µg/mL PKCδ, 50 mM Adenosine triphosphate ATP, and incubated at different time intervals. (**a**) 3 h incubation; (**b**) 6 h incubation; (**c**) 12 h incubation; (**d**) 24 h incubation. Reproduced with the permission of [59].

In 2016, we published a pre-capillary CE-LIF assay for the inhibitor screening of protein kinase C [59]. Our method used FITC labeled ERK peptides F-ERK and P-F-ERK (Extracellular signal Regulated Kinases) to act as Protein Kinase C δ substrates and the enzymatic reaction was conducted in a cellular system (Figure 4). Prior to the enzyme assay, our method accomplished LODs of 4 and 12 ng/mL for F-ERK and P-F-ERK, respectively. The reproducibility for the two peptides was within 5% and accuracy ranged between 86% and 109%. We then successfully calculated the IC50 values of four inhibitors of PKCδ, including staurosporin, bisindolylmaleimide II, gö6983, and rottlerin. Compared to a commercial PKC ELISA kit, our assay provided the exact quantification and was more adaptable to differing enzyme isoforms. Lee et al. described a pre-capillary CE-LIF method to determine the kinase activity of sphingosine for application in preclinical and clinical trials [100]. Their assay allowed the determination of the in vitro activity of both kinase and phosphates, using purified enzymes. While the traditional platforms to study sphingosine activity, like radiometric assays, contained many drawbacks, such as limited sensitivity, semi-quantitative results, poor resolution, and being time-consuming, the CE-LIF-based assay offered a quantitative tool, high sensitivity, and a robust and straightforward method, which was amenable for the study of enzymes of interest in both cell biology and clinical medicine.

DNA demethylation is essential for organism survival and the enzymes that catalyze the reaction have been studied in several bodies of research. However, most approaches investigated in vitro only indirectly via the detection of coproducts. Karkhanina et al. described a method to directly measure the formation of the demethylated DNA product, using a 15 nt long one-base methylated substrate, with the separation only taking 10 min [101]. While separating DNA typically uses the gold standard technique slab gel electrophoresis, using such traditional methods failed to perform, due to the lack of difference in the lengths and conformation of the product and substrate. However, CE successfully achieved well-resolved peaks of both the product and the substrates.

Histone deacetylation plays a vital role in gene expression, and aberrant transcription due to mutated genes that encode histone deacetylaes (HDAC) is a hallmark in the onset progression of cancer [102,103]. Hence, inhibitors of HDACs have emerged as a new class of drugs for the treatment of cancers, because of their effects on tumor suppression, cell growth, and cell survival. Recently, Zhang et al. established a rapid and cost-effective CE-LIF based method, employing a 5-carboxyflyrescein labeled peptide with an acetylated lysine residue as the substrate of HDAC1 to screen the HDAC inhibitors from 38 purified natural products [104]. Their calculated IC50 value for a well-known HDAC inhibitor—suberoylanilide hydroxamic acid SAHA—was consistent with that of the literature. From the screened products, luteolin was identified as an HDAC inhibitor. The method provided herein strengthened the ability of CE-LIF as a universal approach for the screening of many other kinds of enzyme inhibitors.

**Figure 5:**
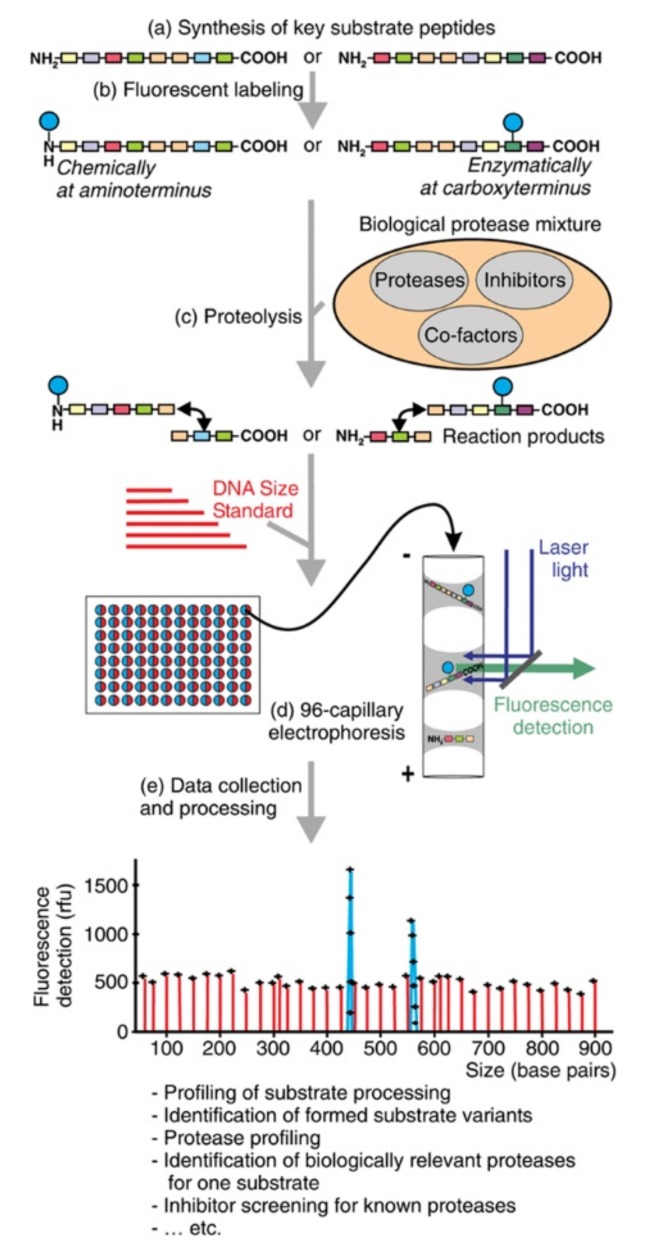
Microfluidic devices used for parallel electrophoretic enzyme assays. (**a**) Design of microfluidic network containing 16 parallel separation channels, (**b**) design of a 36-channel network, (**c**) photograph of a finished 36-channel chip, (**d**) bright-field image of the detection area on the 36-channel chip, (**e**) repetitive units of the microfluidic network. The Electrokinetic injection procedure is described in experimental section. Reproduced with the permission of [105].

Picard et al. introduced a platform for the profiling of multiple proteolytic activities, using a fluorescent-labeled substrate assaying in the absence and presence of protease inhibitors [105] (Figure 5). Using a commercially available 96-channel capillary DNA sequence, coupled with CE-LIF, they successfully demonstrated the monitoring, classification, and inhibition of multiple proteolytic activities acting on the model peptide—an amino acid sequence of mouse granulocyte chemotactic protein-2. Numerous biological protease mixtures, including proteases from tumor cells, neutrophil granulocytes, and plasma, were studied. Although their method could be a high throughput starting point to investigate relevant proteases in cells and in vivo, a critical drawback is that the peptide model studied may not reflect the exact conformation within the context of the protein, therefore implying a substantial impact of the capability of proteases to cleave peptide compared to protein-based substrates.

In 2018, Fayad et al. successfully demonstrated a pre-column CE-LIF assay to study the bioactivity of multiple enzymes, including hyaluronidase, elactase, and collagenase, in search of active cosmetic ingredients. By using a double detection besides LIF, another detection system, termed high-resolution mass spectrometry (HRMS), was connected in series to ensure the simultaneous analysis of three enzymatic reactions. All substrates and products were well defined in the less than 10 min run, with excellent limit of quantification LOQ (<5 nM) and good peak symmetry and efficiency, sufficient repeatability for intra-day and inter-day analysis (RSD < 4.5%) [106]. Prior to this work, the same group introduced a simple electroporation technique to destroy microalgae membranes to extract several amino acids in Dunaliella salina green algae, which were later analyzed by CE-LIF [107].

The compilation of using pre-capillary CE-LIF based in enzyme’s kinetics, drug metabolism, or drug screening is listed in Table 2.

### 4.3. On-Column (In-Capillary) Enzymatic Assays

Bao and Regnier first pioneered the use of a capillary as a micro-reacting system for enzyme reactions [127]. This method was later termed electrophoretically mediated microanalysis (EMMA), or an on-column enzymatic assay. As suggested by the name, this technique integrates all reactions and separating steps into a single column, leading to further automation and extremely small consumption of enzymes, substrates, and cofactors. This approach is especially attractive where no sample workup is necessary and a high degree of miniaturization is achievable. In general, in-capillary assays encompass two modes to load enzyme and substrates, the continuous mode (long contact mode) and the plug–plug EMMA (short contact mode) [128]. In the continuous mode, the entire capillary is filled with either enzyme or substrate. Consequently, the second reactant is flushed through the channel as a plug. In the classical plug–plug EMMA, the enzyme and substrate are introduced as consecutive plugs, and the enzyme reaction takes place upon the application of an electric field. One drawback of EMMA is the incompatibility between the background electrolyte required for the separation and the enzyme reaction buffer. This shortcoming is not usually a big challenge, since in many cases background electrolytes consume similar buffers, which are used for sample preparation. However, to address this incompatibility, an additional plug of incubation buffer is injected into the system. This mode is named “partial filling mode” [129].

### 4.4. Application

The use of the EMMA approach has expanded in recent years for studies on enzyme activity and kinetics, inhibitor screening, substrate determination, and drug metabolism. In particular, the application of LIF detection has increased, especially in clinical or analogously oriented studies.

**Figure 6:**
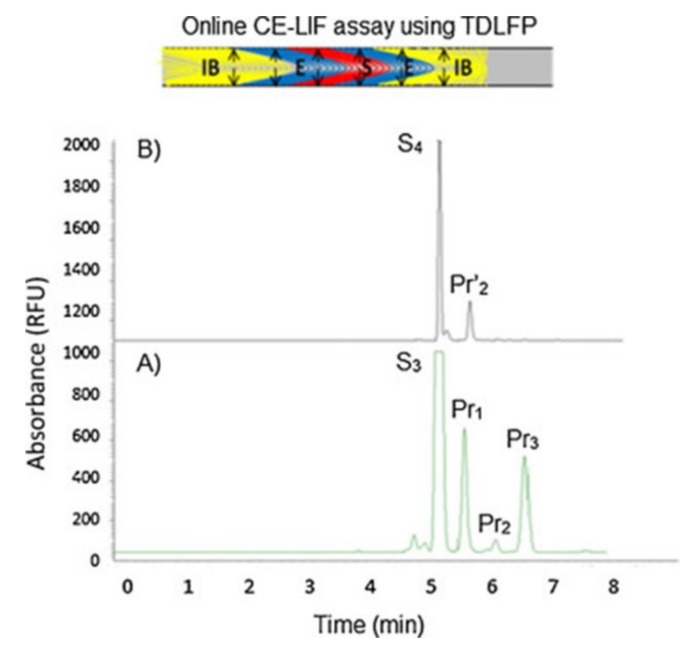
Electropherograms obtained by CE-LIF analysis for (**A**) S_3_ or 5-FAM-Ala-Ala-Ala-Phe-Tyr-Asp-OH and (**B**) S_4_ or 5-FAM-Arg-Glu-Ala-Val-Val-Tyr-OH hydrolysis by HNE. Reproduced with the permission of [108].

Fayad et al. reported an online EMMA-CE-LIF assay to evaluate the kinetic constant of a novel substrate of human neutrophil elastase (HNE), an enzyme responsible for skin aging and involved in the development of chronic obstructive pulmonary disease in non-small cell lung cancer progression [130,131]. Based on short-end injection, using transverse diffusion of laminar flow profiles (TDLFP) to mix the reactants, the analysis time was shortened to only 7 min [108]. The TDLFP mixing technique has also been used to study many enzyme activities [132,133,134]. HNE activity was assessed using both UV and LIF detection modes (Figure 6). The higher sensitivity obtained from LIF (almost three-fold in magnitude at a few nM LOD) is proof of the concept that LIF coupled with CE is economical and highly selective. The same group later developed another EMMA-CE-LIF-based method to study the inhibition of HNE [27]. For this, they utilized a complimentary technique termed microscale thermophoresis (MST), coupled with CE-LIF, to efficiently monitor the enzyme-inhibitor affinity. Benefiting from the previously published work, this assay to screen HNE inhibitors reached a high level of LOQ (~3 nM) with no pre-concentration steps required.

Coyle’s group developed a method to study phosphatases from marine bacteria whose enzymatic functions are of importance to the mobilization, transformation, and turnover of compounds in aquatic environments [109]. Based on the precedence of previous work [135], they determined the alkaline phosphatase’s kinetics in four marine proteobacteria isolates, using a fluorescently labeled substrate 3-o-methylfluorescein phosphate cyclohexylammonium salt MFP. Without significant modifications, the CE-LIF online approach provided more information about enzyme diversity and could be applied to study the phosphatases in various organisms. However, challenges remain for the method, including the identification and characterization of enzymes which have the same electrophoretic mobility.

Diabetes is one of the most common metabolic disorders worldwide, characterized by high blood sugar levels in patient serum [136,137]. Due to its lethal complications, many research groups have been extensively seeking to develop fast, accurate, and reliable methods to read and/or monitor glucose. Recently, Guan et al. introduced an ultrasensitive analysis of glucose in serum, using a CE-LIF on-column enzymatic assay [110]. The reaction between glucose oxidase (GOx) and horseradish peroxidase (HRP) released hydrogen peroxide (H2O2), leading to the activation of a fluorogenic reagent named 2-[6-(4’-amino) phenoxy-3H-xanthen-3-on-9-yl] benzoic acid APF to form a highly fluorescent product, which was later electrophoretically separated from unreacted APF by the LIF detection. Their proposed method allowed the detection of glucose in real samples down to 10 nM, and RSD values lower than 3.5%.

In addition, other components of biological fluids could be targets using the EMMA-CE-LIF platform. The same group reported this method in the use of determining total cholesterol in human plasma [111].

Nicotinamide adenine dinucleotide (NAD+) and its reduced form NADH act as two coenzymes that are involved in cellular energy metabolism, including glycolysis and oxidative phosphorylation. The conversion of the two enzymes, or the NADH/NAD+, ratio reflects the cellular metabolic status, thus responding to environmental stimuli. Xie and colleagues developed an in-capillary assay to detect NAD+ and NADH contents of a single cell, by coupling an enzymatic cycling reaction with CE-LIF [112]. NAD+ is reduced to NADH in one enzymatic reaction, and in return NADH is oxidized to NAD+ with the production of a fluorescent product [138,139]. As the cycle goes on, with the formation of a fluorescent product, resorufin, the accumulation rate is therefore correlated to the NAD+/NADH ratio in the system. Xie reported the LOD for NAD+ using on-line capillary assays being as low as 0.2 nM, with the reproducibility (RSD) of 5%, which is much more sensitive than that of concurrent methods available for the determination of NAD+ and NADH in cells and tissues, such as fluorescence imaging, enzymatic assay, or HPLC.

The recent EMMA-CE-LIF methods are listed in Table 2.

### 4.5. Chip-Based Enzyme Assays and Application in CE-LIF Systems

In general, the application of microchip devices in capillary electrophoresis has been targeted to acquire the miniaturization and portability features. To join in LIF mode, a significant reduction in the size of the auxiliary apparatus is necessary to achieve such a sophisticated system. Initially, since the laser module is bulk equipment, microchips have acted as a sample preparation tool in need of pre-concentration or purification. However, several research groups have been seeking methods to integrate the whole fluorescence detector inside the microchips, a new concept known as “lab-on-a-chip”. The most common strategy is to embed optical fibers inside the micro devices to bring the light in and out of the detection unit. Various designs have been introduced with the optical fiber inserted onto the microchip. Among them, optical micro lenses have become a trend, using different materials and techniques. For examples, polymer micro lenses have been utilized to focus light inside the microfluidic channel and increase the excitation of the fluorescent tags. To apply this strategy and aim for high-throughput in design, Guestchow’s group fabricated polydimethylsiloxane (PDMS) on a chip device [140]. By taking advantage of the intrinsic properties of glass (hydrophilic) and PDMS (hydrophobic), the extraction of droplets from the segmented flow was simplified. The segmented flow sample streams were coupled with hybrid PDMS-chip glass and rapidly analyzed by microchip electrophoresis with a dual beam laser-induced system. A total of 160 test compounds as potential inhibitors of protein kinase A were subsequently screened and each sample generated two droplets, thus allowing approximately six injections per sample. Including controls (negative and positive), a total of 168 samples were analyzed in approximately 12 min. Of the screened compounds, 25 potential enzyme inhibitors were identified, with the IC50 of two inhibitors calculated.

Microchips in CE usually use a very short separation distance. Therefore, it is crucial to effectively deliver narrow and reproducible sample plugs into the separation channels. Optically gated sample introduction is an alternative injection method from the commonly used T-type injection. In this injection mode, fluorescently labeled analyte is electrophoresed through the separation section. A laser is split into two beams and focused on the two points of the separation section. One beam (the probe beam) has less laser power and is utilized for LIF detection. The other beam (the gating beam) has higher laser power and is used to perform the sample introduction by time-discriminated photobleaching of the sample. For instance, Gong et al. introduced a microfluidic device-based enzyme assay using beta-glucuronidase as the enzyme and a fluorescein di-(beta-D-glucuronidase) as the substrate model [141]. Their device was coupled with a dual-beam LIF detection, where the fluorescent signals were recorded with two independent LIF channels capable of being aligned to various positions on the device. Consequently, the enzymatic product concentrations in the manner of time incubation were measured, the Michalis constant was determined, and inhibition assays were carried using a competitive inhibitor to calculate the IC50 values of promising inhibitors.

In the study of Belder’s group, they demonstrated a rapid CE-chip-based assay to investigate the deacetylation of acetyl-lysine residues by sirtuins (SIRTs) enzymes [142]. Their microchip design consisted of a microfluidic separation structure integrated with a serpentine micromixer. 9-fluorenylmethoxycarbonyl (Fmoc)-labeled tetrapeptide derived from p53 was used as the SIRT substrate. The substrate and enzyme were mixed in the reaction channel and subsequently transferred to the separation channel by an electrophoretic pinched injection. The SIRT inhibitors were screened, with the results in alignment with the literature. The total time, including incubation, chemical reaction, and analysis, was about 30 min, which was shorter than the previously published techniques [143].

Besides reaction in solution, enzymes can also be immobilized to solid supports, known as immobilized enzyme reactors (IMERs), which are incorporated into CE-chips to further utilize the EMMA technique in a miniaturized system. Reviews were published using IMERs in proteomics [144] or microfluidic process reactors [145] and field-analysis [84]. Microchip CE has employed IMERs in its offline approach, in combination with the LIF detection mode. For example, Qiao published a series of microchip CE-LIF assays to study the enzyme activities of L-asparaginase [146,147]. Their results demonstrated a promising therapeutic protocol for acute lymphoblastic leukemia.

## 5. Conclusions and Perspective Outlook

This review discussed the application of CE-LIF as a tool to develop immunoassays and enzyme assays and its application in the last decade. Due to the increasing demand in biological, clinical, and pharmaceutical research, the use of CE-LIF has been expanding rapidly, because of its ultra-sensitivity and selectivity. The instruments and method are continually changing to improve the efficiency of the analysis, as well as bridging the application of CE to broader spectrums of analysis. Scientists are extensively seeking to modify the system by introducing new materials for capillary column modification, or analyte derivatization to maximally enhance the analytical performance. Emerging as a new trend, nanoparticles (NPs) with a size of less than 100 nm have attracted much attention for a widespread range of applications, due to their unique physicochemical characteristics and facile surface modification. Among various NPs, employment of magnetic beads as solid support for immunoextraction has been seen in many reports [148,149,150,151,152]. The number of studies and applications of NPs as independent surrogates or in coupling with the microfluidic process in immunoaffinity and the enzymatic assays is continuously increasing, with numerous papers dealing with NPs in the CE system.

To improve the performance of complex biological and biomedical samples, multidimensional separation has become a tendency, where two or more orthogonal displacement mechanisms are combined in CE systems. Especially with regards to fluorescence detection, multiple dimensions have helped overcome the matrix interferences, thus significantly improving the sensitivity. Together with the combination of microfluidic devices, CE-LIF has enhanced efficiency, as well as allowing the multiplexed separation of analytes. An integrated 2D, 3D, or more dimensional system in the future will make CE separation a portable and universal tool of practice in medicine, pharmaceuticals, environmental science, and food science.

LIF detection has been well established for decades in the capillary electrophoresis field. However, in an attempt to reduce the cost of expensive laser modules and avoid stability issues in the baseline (especially in the UV range), researchers have been looking for alternative light sources for LIF. CE-LEDIF has been a replacement candidate, which was discussed briefly earlier due to the various emission wavelengths that LED sources can offer. In terms of detection limit, it is still controversial compared to LIF, however, in the near future, LEDIF can possibly replace LIF detection.

We discussed herein the CE-LIF immunoassays and pointed out the advantages, disadvantages, and points to consider of each format. For immunoassays, competitive homogenous binding assays are still the most popular of the CE-LIF based immunoassay methods. We also included a discussion of the enzymatic reaction. Both kinds of reaction have been applied successfully to capillary electrophoresis. Of enzyme assays, offline methods are easy to perform and manage. With a combination of EMMA, IMERs, and multidimensional channels, we attempted to bring to light the immunoassay and enzyme assay point of view to the analytical scientists, and highlight the opportunity to harness this powerful technique within analytical chemistry. Table 3 lists the common advantages and disadvantages offered by CE-LIF for these bioassays.

Intriguingly, chip devices have inevitably proved their capacity to incorporate with CE to become a fast, high throughput, and automated miniaturization system. We covered the application of microfluidic devices in both categories to showcase the volatility and ease of use upon the demanding and challenging requirements of industrial purposes. At its core, CE-LIF can become a prognostic or diagnostic tool in multiple clinical fields.

## Figures and Tables

**Table 1:** Recent CE-LIF immunoassays.

Analyte	Format	Labeled	LOD	Ref.
CCP peptides	Competitive	FITC	4 ng/mL	[60]
Alpha-fetoprotein	Non-competitive	FITC	0.05 μg/mL	[64]
DNA fragments	Non-competitive	Alexa Fluor 546	0.3 nM	[66]
Thrombin	Non-competitive	TMT aptamer	0.2 nM	[66]
Thrombin	Non-competitive	TMT aptamer	0.2 nM	[67]
Glucagon	Non-competitive	6-FAM aptamer	6. 0 pM	[68]
amyline	Non-competitive	6-FAM aptamer	40 pM	[68]
IgE	Non-competitive	5-FAM	250 pM	[69]
human immunodeficiency virus reverse transcriptase	Non-competitive	5-FAM	100 pM	[69]
PDGF-BB	Non-competitive	5-FAM	50 pM	[69]
Recombinant human erythropoietin-α	Non-competitive	FITC	0.2 nM	[70]
human serum albumin	Competitive	FITC	1.34 × 10^−7^ M^−1^	[72]
norfloxacine	Competitive	FITC	0.005 μg/L	[73]
carbaryl	Competitive	FITC	0.05 ng/mL	[74]
chloramphenicol	Competitive	FITC	0.0016 μg/L	[75]
receptor beta- pdgf	Competitive	6′-FAM	3 nM	[76]
receptor alpha- pdgf	Competitive	6′-FAM	0.5 nM	[76]
testosterone	Competitive	FITC	1.1 ng/mL	[77]
chloramphenicol	Competitive	FITC	7.6 × 10^−9^ g mL^−1^	[78]
glucagon	Competitive	FITC	5 mM	[79]

**Table 2:** Recent CE-LIF enzyme assays.

Enzyme	Substrate	Mode	Note	Ref.
neutrophil elastase	5-FAM-labeled peptides	on column	enzyme activity and inhibitor screening	[27]
protein kinase C	fluorescent-labeled peptide	off column	inhibitor screening	[59]
Sphingosine kinase	Fluorescein-labeled sphingosine	off column	kinase and phosphatase activity	[100]
AlkB	fluorescently labeled 15-nucleotide-long single-base methylated DNA substrate	off column	demethylation of DNA	[101]
histone deacetylase 1	5-carboxyfluorescein-labelled peptide	off column	inhibitor screening	[104]
Proteases	Fluorescence-labeled peptide	off column	proteolytic processing	[105]
Hyaluronidase, elastase and collagenase	FAM-peptides	off column	enzyme kinetics and plant substrate	[106]
Human neutrophil elastase	5-carboxyfluorescein (5-FAM) peptide	on column	enzyme kinetics, substrate study	[108]
alkaline phosphatase	MFP cyclohexylammonium	on column	enzyme kinetics and activity	[109]
glucose oxidase	2-[6-(4′-amino) phenoxy-3H-xanthen-3-on-9-yl] benzoic acid (APF)	on column	glucose determination, inhibitor screening	[110]
cholesterol oxidase	2-[6-(4′-amino) phenoxy-3H-xanthen-3-on-9-yl] benzoic acid (APF)	on column	cholesterol measurement	[111]
Lactate dehydrogenase	lactate	on column	enzymatic cycling reaction	[112]
recombinant human arylsulfatase	glycosaminoglycan	off column	enzyme kinetic, natural substrate	[113]
beta-glucosidase	Fluorescein mono-beta-D-glucopyranoside	off column	enzymatic activity	[114]
Calcineurin	Fluorescence-labeled 19-amino acid	off column	kinase activity	[115]
ATP	BODIPY FL EDA	off column	enzyme activity	[116]
Lysine decarboxylase	Lysine	off column	enzyme activity	[117]
oxygenases	AlkB	off column	inhibitor study	[118]
l-Asnase	FITC amino acids	off column	FITC amino acids	[118]
d-amino acid oxidase	d-amino acids	off column	enzyme activity	[118]
Signal peptidases	proprietary fluorescent-labeled substrate	off column	inhibitor screening	[119]
tyrosine kinase	fluorescence-labeled polypeptide substrate	off column	kinase activity, inhibitor screening	[120]
horseradish peroxidase	thyroxine, triiodothyronine, thyroid-stimulating hormone	on column	hormone study	[121]
recombinant GFP	thrombin	on column	enzyme activity	[122]
beta-galactosidase	resorufin-β-D-galactopyranoside	on column	Single molecule enzymology	[123]
protein farnesyltransferase	fluorescently labeled pentapeptide, farnesyl pyrophosphate	on column	inhibitor screening	[124]
alkaline phosphatase	AttoPhos	on column	enzyme inhibitor study	[125]
alkaline phosphatase	disodium phenyl phosphate	on column	enzyme catalysis	[126]

**Table 3:** CE-LIF based assay advantages and disadvantages.

Advantages	Disadvantages
-High sensitivity, high speed -High reproducibility -Small number of samples required -Minimal preparation time -Easy automation, high throughput for profiling of complex biological samples -Possible on-column concentration -Highly efficient multidimensional separation	-Derivatization required -Instability of the laser power -Excitation range is limited -No standardized method
